# A cost–benefit analysis for use of large SNP panels and high throughput typing for forensic investigative genetic genealogy

**DOI:** 10.1007/s00414-023-03029-7

**Published:** 2023-06-21

**Authors:** Bruce Budowle, Andrew Arnette, Antti Sajantila

**Affiliations:** 1 Department of Forensic Medicine, University of Helsinki, Helsinki, Finland; 2 Radford University Forensic Science Institute, Radford University, Radford, VA USA; 3 Department of Business Information Technology, Virginia Tech, Blacksburg, VA USA; 4 Forensic Medicine Unit, Finnish Institute for Health and Welfare, Helsinki, Finland

**Keywords:** SNPs, Forensic investigative genetic genealogy, Cost–benefit analysis, Database, Tangible benefits, Intangible benefits

## Abstract

Next-generation sequencing (NGS), also known as massively sequencing, enables large dense SNP panel analyses which generate the genetic component of forensic investigative genetic genealogy (FIGG). While the costs of implementing large SNP panel analyses into the laboratory system may seem high and daunting, the benefits of the technology may more than justify the investment. To determine if an infrastructural investment in public laboratories and using large SNP panel analyses would reap substantial benefits to society, a cost–benefit analysis (CBA) was performed. This CBA applied the logic that an increase of DNA profile uploads to a DNA database due to a sheer increase in number of markers and a greater sensitivity of detection afforded with NGS and a higher hit/association rate due to large SNP/kinship resolution and genealogy will increase investigative leads, will be more effective for identifying recidivists which in turn reduces future victims of crime, and will bring greater safety and security to communities. Analyses were performed for worst case/best case scenarios as well as by simulation sampling the range spaces with multiple input values simultaneously to generate best estimate summary statistics. This study shows that the benefits, both tangible and intangible, over the lifetime of an advanced database system would be huge and can be projected to be for less than $1 billion per year (over a 10-year period) investment can reap on average > $4.8 billion in tangible and intangible cost-benefits per year. More importantly, on average > 50,000 individuals need not become victims if FIGG were employed, assuming investigative associations generated were acted upon. The benefit to society is immense making the laboratory investment a nominal cost. The benefits likely are underestimated herein. There is latitude in the estimated costs, and even if they were doubled or tripled, there would still be substantial benefits gained with a FIGG-based approach. While the data used in this CBA are US centric (primarily because data were readily accessible), the model is generalizable and could be used by other jurisdictions to perform relevant and representative CBAs.

**Supplementary Information:**

The online version contains supplementary material available at 10.1007/s00414-023-03029-7.

## Introduction

Next-generation sequencing (NGS), also known as massively sequencing, offers several advantages over capillary electrophoresis (CE)-based methods for forensic genetic analyses, which include increased sensitivity of detection, higher resolution, and higher throughput [1–10]. This technology has come to fruition with commercially validated kits for autosomal short tandem repeat (STR) analyses, as well as Y-STRs that also can generate investigative leads [11–19], whole genome mitochondrial DNA sequencing [20–23], and more recently with kits or panels for a large number of single nucleotide polymorphisms (SNPs) [24–28]. Indeed, dense SNP analyses have been the genetic component of an explosion of forensic investigative genetic genealogy (FIGG) cases to help solve cold cases, such as identification of the Golden State Killer [29], to support current investigations, such as the recent murders at the University of Idaho [30], missing persons identifications (e.g., see [31]), and internationally [32], as well as facilitate postconviction exonerations (e.g., Ricky Davis, [53]). FIGG combines genome-scale DNA testing and genealogical surveying to determine biological relationships (i.e., kinship) between individuals to indicate a potential source of biological evidence. Studies have shown that relatives as distant as ~ 9 generations may be associated using dense SNP data [34–40], although a distance of 3rd and 4th degree relatives is more practical for FIGG. The genetic power of FIGG was bolstered initially by using SNP microarrays, such as the Illumina Infinium CytoSNP-850 K BeadChip and Infinium Global Screening Array (GSA), each containing more than 600,000 SNPs distributed across the nuclear genome. The costs of generating reference sample profiles were relatively low, but the relatively large amount of DNA quantities required greatly limited the application of microarrays for analysis of forensic samples [41]. However, one benefit of microarray data is that there are at least 40 million dense SNP profiles (i.e., reference samples) in direct-to-consumer companies such as Family Tree DNA, My Heritage, 23andMe, and Ancestry.com [42], which are substantial genome scale data resources if they were made accessible for forensic investigations. A subset of these typed individuals (> 1.4 million) has opted to take their data from the direct-to-consumer services, voluntarily upload their profiles to GEDmatch and allow them to be used for violent crime and unidentified human remains investigations using FIGG [43]. Family Tree DNA also has enabled utilization of its database for a subset of violent crimes and missing person investigations [44]. Utilization of these databases for lead generation has led to some impressive identifications of the sources of forensic biological evidence [29–33].

Because of its increased sensitivity of detection compared with microarrays and CE, NGS allows for dense SNP analyses on low-quantity and low-quality DNA samples [8–10, 45]. Recently, there have been studies demonstrating that targeted SNP panels (5-10 K SNPs; termed herein as large SNP panels) can provide kinship associations up to 4th degree (and with some degree of accuracy at the 5th degree) relatives [24, 25]. Additionally, because a large battery of markers is analyzed, missing person cases may not require reference samples from specific lineage relatives (such as a maternal relative for mitochondrial DNA analyses or a paternal relative for Y chromosome STR typing); indeed, a single family member reference, regardless of maternal or paternal lineage, may be sufficient for supporting or refuting whether a particular human remains belongs to a pedigree. Thus, the genetic analysis component of FIGG is notably attainable and could be implemented into the operation-oriented casework laboratory making such analyses no longer the sole purview of large genome centers or private entities.

With the advantages of NGS, seemingly the technology would be embraced by the law enforcement and forensic science communities. The former has proceeded forward making use of private entities to obtain FIGG services. The latter has been slow to embrace dense or large SNP technologies due to real and/or perceived impediments to implementation. These impediments are due partly to budgets, resources, training, labor and time, and due partly to legislation, policies, and privacy issues [47]. The policy and privacy issues, which are important, are not considered herein, although it is noted that, for example, Maryland [47] and Utah [48] already have established policies to employ FIGG. Herein, the issues associated with cost are addressed.

One hurdle for implementation in the crime laboratory often espoused is that NGS is more costly than CE. That assertion at first glance may appear to be a reasonable assessment. Assuming that both systems (CE-based and NGS-based) were properly supported within an effective infrastructure, it would appear that the cost of NGS to generate a large SNP profile is greater than that generating a STR profile by CE or NGS. However, this view is narrowly focused and does not necessarily consider the system-level impact this technology can have within the criminal justice system and on society as a whole.

First, the cost of generating a STR profile by NGS (while not the focus of this study) already is comparable on a per sample profile. For example, consider the ForenSeq MainstAY Kit (Verogen) at the cost of $2390 for processing 96 samples. Thus, the library preparation cost per sample would be ~ $25. Adding in the cost of a standard MiSeq FGx Reagent kit (at $1500) for sequencing the overall reagent cost per sample, when processing 96 or 24 samples simultaneously, would be ~ $40 and ~ $88, respectively. This cost is comparable to that of generating a STR profile by CE, assuming that the amount of labor to process the sample is similar to that of CE typing or automated workflows that are employed. The $88/sample cost may be perceived to be on the higher end but given the versatility of NGS to type far more markers per sample per analysis, the ability to reduce the number of samples simultaneously sequenced in a run to gain read depth, and most importantly the ability to generate additional investigative leads should justify the modest increased cost.

Second, and the focus of this study, a better perspective for considering whether to move forward with dense or large SNP analysis by NGS should be the cost in light of the benefits that may be provided based on a systems approach [49–53]. As such, this study entails evaluating whether the potential performance and capabilities of NGS (i.e., increased sensitivity, throughput over current technologies, and straight forward SNP analyses), enhancements through genealogy enable tangible and intangible savings, and benefits to victims, families, and communities, as well as government and personal budgets on a routine-use basis, may justify the higher upfront laboratory costs. Even more so, do the costs versus benefits with NGS/FIGG warrant a mandate to move the technology forward as has been done with other DNA initiatives, such as increasing the CODIS core STR markers required for sample typing [54]? For example, consider the current profile upload rate to the National DNA Index System (NDIS) of CODIS and the hit rate with searches in NDIS associated with sexual assault kit analyses are 41.6% and 47.3%, respectively (see SAKI data [55] presented in the “Results and discussion” section for details on these values). These data suggest that the overall hit rate for all sexual assault kits completed is ~ 19.7% (i.e., 0.416 × 0.473). If a technology, such as large SNP panel analysis via NGS, provided an increased upload rate, for example, hypothetically and modestly to 45% and an increase in investigative leads to around 80%, the overall success rate for completed kits could increase to 36%, almost doubling the number of potential of leads. This benefit to sexual assault victims (and families and communities) alone would make it hard pressed to argue against using FIGG, even if it costs more than current STR analysis technologies. It would be incumbent upon the investigative and forensic laboratory systems to pursue increased laboratory budgets to enable the use of NGS to support FIGG investigations, if there were real benefits to society. A prospective cost–benefit analysis (CBA) could help an agency determine if and support that the technology enhancement can have a large impact on the criminal justice system and society as a whole (e.g., the agency, other agencies, victims, families, communities, and taxpayers).

Budowle et al. [49, 50] recently showed using a CBA model (both with static bounding values and with a Monte Carlo simulation) that replacing lower performing cotton swabs with higher performing, higher cost nylon 4N6FLOQSwabs® (COPAN Italia, SpA) is justifiable and a highly beneficial investment. A similar CBA can be performed for assessing NGS and FIGG. The ForenSeq Kintelligence Kit (Verogen) (referred to as Kintelligence kit herein) can be used to model the cost incurred, as this kit is the only commercially available one with a large number of targeted SNPs (i.e., 10,230 SNPs) [26, 28]. A similar approach for whole genome sequencing (WGS) with low pass coverage (to reduce WGS costs) could be entertained as it conceivably could yield SNP and STR data [56]. However, the costs conceivably could be more expensive than that of the Kintelligence Kit and other quality issues would have to be addressed as well [28] and, importantly, the WGA approach may be less conducive to productization and implementation in operational public laboratories. The Kintelligence Kit is deemed a better model as it is more likely to be implemented in a forensic laboratory than would be WGS, would have better depth of coverage per locus, be more robust for typing when samples are comingled with microbial DNA, support a broad range of degraded and chemically-contaminated forensic samples, and still would generate kinship relationship associations for most scenarios [26, 28].

The logic, strengths, and limitations of this CBA are described herein. For simplicity, initially, the highest and lowest benefits were calculated statically to provide insight on the bounds of what could have been gained over the lifetime of the current national DNA database operations, i.e., CODIS, if a SNP-based approach had been in operation instead, as well as annually for insight on what can be gained going forward if a FIGG analytical/investigative approach was pursued. Since annual projections are more informative, best estimates also were calculated using Monte Carlo simulations. All analyses were performed on three crime categories — sexual assault, murders, and all other crimes combined. For missing person cases, there are little or no tangible and intangible costs reported for humanitarian efforts that can be used as an input to the model. Therefore, only a comparison of reagent costs was carried out. Lastly, the costs to build the operational forensic DNA laboratory and populate a database over a prolonged ten-year time frame were estimated to determine if the cost-benefits support the estimated expenses. Overall, the tangible and intangible cost savings are substantial and support upfront infrastructural investments in the public laboratory system and developing a large SNP panel-based database system. Moreover, the huge potential to prevent some people from ever becoming victims (sexual assault, murder, property crime, etc.); the gain in resolution for some victims, families, and communities; the increased safety and security; and an increase in confidence from the public should be sufficient to warrant an investment in this technology for the operational laboratory and implementing a supporting database system.

## Materials and methods

This CBA follows the logic that an increase of DNA profile uploads to a DNA database due to a sheer increase in number of markers and a greater sensitivity of detection afforded with NGS and a higher hit/association rate due to large SNP/kinship resolution and genealogy will increase investigative leads, will be more effective for identifying recidivists which in turn reduces future victims of crime, and will bring greater safety and security to communities. It should be noted for this study the terminology “hit” and “hit rates” may be used throughout for facilitating communication and assessment for both the STR/CE-based current government-maintained database approaches and for NGS/SNP/FIGG database approaches, which currently are maintained by private entities. A hit in the former approach typically is a one-to-one direct comparison leading directly to a potential source of biological evidence. In contrast, in the latter system, currently, an association or better stated an “investigative lead” is made between the unknown donor of biological evidence and a relative via indirect (or kinship) comparisons, and the relative(s) is not a person of interest. Thus, the use of the term “hit” herein, when used, is not the same outcome between these two analytical/database approaches. This study compiled forensic cases into four categories: (1) sexual assault, (2) murder, (3) all other violent and property crimes (grouped for illustrative purposes and simplification), and (4) missing persons. It should be noted that FIGG in the USA currently is used for investigations of violent crimes, such as sexual assault and homicide, and for missing persons identifications, but not for crimes such as property crimes. However, the SNPs in the Kintelligence panel were searched against ClinVar and found not be associated with medical/health information; thus, the risk to privacy is reduced greatly compared with genome wide SNP data [26]. Therefore, for the study herein, it would seem reasonable to treat these large SNP panel data in a similar fashion as are STR data and assume the approach can and should be used to assist in developing investigative leads for any crime in which biological evidence may be probative. For this thought exercise, a large SNP infrastructure within a national laboratory network and database system is assumed (i.e., properly stocked reference database representative of major populations of a jurisdiction, laboratories fully operational, and all protocols and policies for analysis are in place), in other words, a database-supported system as mature as current ones, such as CODIS. The data used to estimate parameters are based on the most recent statistics reported at NDIS (National DNA Index System, October 2021) [57], in the Sexual Assault Kit Initiative (SAKI) [55], number of requests submitted to government laboratories for biological examinations [58], on tangible and intangible costs associated with crime reported by Miller et al. [59], on additional investigator time and costs [33, 60], on property crime and DNA typing successes reported by Roman et al. [61], on serial murder estimates [62–64], and on outcomes of investigations reported in the FBI’s Uniform Crime Report (UCR) [65].

To the best of the authors’ knowledge, this CBA is the first one performed for NGS, a large SNP panel, and FIGG. Therefore, substantial detail is provided in the text (as well as captured in the tables) on the modeling process, estimates, and calculations. As such details at times may be difficult to follow, the general approach to the CBA model used herein is listed in Table 1 to present a simplified, concise version of the process for the readership.

**Table 1:** General steps of the model used to determine cost-benefits of using a large SNP profiling approach^a^

Steps	Actions
1	Identify database source(s) of forensic sample profiles
2	Separate forensic profiles with hits (i.e., hit category) and forensic profiles that have not yielded hits (i.e., no hit category)
3	Determine overall hit rate
4	Estimate range of proportion of profiles associated with each crime category considered in analysis (i.e., sexual assault, all other crime categories combined, murder)
5	Estimate range of increase in number of typeable (i.e., uploadable) profiles based on increased sensitivity of detection range
6	Estimate association or investigate lead rates based on large SNP profile analyses
7	Estimate recidivism rate ranges
8	Estimate range of number of victims that could be reduced based on recidivism rates and early detection of serial perpetrators
9	Estimate tangible and intangible costs associated with type of crime
10	Add additional costs (in this analysis is increase in police investigation hours)
11	Calculate cost-benefits savings based on increase in typeable samples, increase in investigative lead rate, reduction in number of victims and tangible and intangible costs per type of crime

^a^These are general steps and can vary in detail per analysis described below

Initially, descriptive statistics were used to derive the cost-benefits at only the lowest and highest input brackets, which are limited samplings of the potential data space. Since any parameter considered could have a range of possible values, Monte Carlo simulations also were performed to sample the range spaces simultaneously to generate best estimate summary statistics. The ranges and base case values of the various inputs that were sampled via simulation are listed in Tables 2, 3, and 4. The Monte Carlo simulation modeling technique allows for an assessment of risk and uncertainty by generating a probability distribution of different outcomes through repeated sampling of random point estimations followed by averaging of results. As some base data are not collected or compiled or are not readily available, ranges of values were used as input for the purpose of estimation. For many of the inputs to the calculations, a triangular distribution model was employed using average, low, and high estimates. Other parameters had only ranges, which were then modeled using uniform distributions. The model was created using Frontline Systems Analytic Solver Platform (FrontlineSolvers®) [66], with 1000 trials generated, each independently sampling from the eighteen probability distributions defined within the model.

**Table 2:** Ranges for input values for sexual assault cases^a^

	Min	Max	Base case
Tangible cost	$5000	$10,000	$7419
Intangible cost	$100,000	$165,000	$133,021
Increase of uploads	5%	20%	10%
Upload ratio	41.6%	50%	45%
Total cases^b^	330,000	400,000	330,000
Investigative lead rates	59%	88%	76%
Recidivism/victim reduction rate	30%	67%	67%
Extra hours for investigation	10	50	30
Police hourly rate^c^	$24	$71	$46

^a^Ranges based on empirical data and judgmental data based on authors’ experience

^b^Sexual assault cases range from 13.5 to 20% of total cases

^c^Pay rates derived from Nashville, TN (for min), and San Francisco, CA (for max), police salaries

**Table 3:** Ranges for input values for all other violent and property crimes cases^a^

	Min	Max	Base case
Tangible cost	$6000.00	$15,000.00	$8261.00
Intangible cost	$20,000.00	$40,000.00	$30,942.00
Increase of uploads	5%	20%	10%
Upload ratio	50%	60%	55%
Total cases^b^	330,000	400,000	330,000
Investigative lead rates	59%	88%	76%
Recidivism/victim reduction rate	30%	65%	65%
Extra hours for investigation	10	50	30
Police hourly rate^c^	$24	$71	$46

^a^Ranges based on empirical data and judgmental data based on authors’ experience

^b^All other crimes range from 80 to 86.5% of total cases

^c^Pay rates derived from Nashville, TN (for min), and San Francisco, CA (for max), police salaries

**Table 4:** Ranges for input values for murder^a^

	Min	Max	Base case
Tangible cost	$2,000,000	$3,000,000	$2,658,319
Intangible cost	$4,000,000	$6,000,000	$5,150,836
Increase of uploads	5%	20%	10%
Upload ratio	30%	50%	40%
Total cases	6,000	7,100	6,672
Investigative lead rates	59%	88%	76%
Recidivism/victim reduction rate	5%	15%	15%
Extra hours for investigation	10	50	30
Police hourly rate^b^	$24	$71	$46

^a^ Ranges based on empirical data and judgmental data based on authors’ experience

^b^Pay rates derived from Nashville, TN (for min), and San Francisco, CA (for max), police salaries

After the probability distributions were defined, the model was run and distributions of possible outcomes, along with key statistical measures, were created for each of the outcome values of interest, specifically total tangible costs, total intangible costs, and victim reductions for each of the three categories (sexual assault, murder, and other crimes). The histograms show the distribution of possible outcomes, providing more detail than an average or range. Additionally, sensitivity analysis charts were created for each measure in the form of a tornado chart showing the impact of the probability distribution on the outcome values (with all other distributions held at a constant value).

Statistics were compiled and summarized. Explanations for the ranges and support for the values are described in the “Results and discussion” section. If desired, alternative values that one may consider more appropriate can readily be substituted and calculated.

## Results and discussion

This CBA is based primarily on two premises. The first premise is that large SNP analyses via NGS will yield more usable DNA profiles from crime scene samples than current STR analysis via CE technologies which in turn will increase the total number of DNA profiles uploaded to a database per year as well as over the long term. More profiles will increase the number of offender and forensic hits. As stated above, there is strong support that NGS offers greater sensitivity of detection than that of CE. This proposition is intuitively and empirically based. Since detection of STRs is based on sequence and fluorescence and size separation are not employed, amplicons can be designed, where possible, that are shorter in length and of similar lengths among loci. Thus, there is an expectation that amplification efficiency and analysis of degraded samples will improve with NGS analyses compared with CE analyses. As an example, Stephens et al. [19] demonstrated with the ForenSeq MainstAY kit that even at 8 pg (with four replicates) on average 32 autosomal STR loci (61% of all expected autosomal alleles) and 20 Y-STR loci (70% of all expected Y alleles) could be detected. With the Kintelligence SNP panel, the vast majority of amplicons are < 150 bp which also supports efficient amplification and greater success for typing degraded samples. Peck et al. [27] reported a 92.1% mean concordance rate with 50 pg of input DNA (the lowest input amount tested) with the Kintelligence kit. Antunes et al. [28] reported that out of the 10,230 SNPs in the Kintelligence panel > 6900 SNPs were detected at 25 pg of input DNA which are sufficient to potentially estimate 4th degree relationships. Even at 6.3 pg, greater than 2100 SNPs were detected. These numbers of SNPs with such low-quantity input amounts still are quite informative for some casework scenarios, such as direct comparisons and evaluation of first degree genetic associations. Therefore, there is support that the use of NGS and large SNP panels (considered herein) can increase the number of samples that yield information for investigative leads across a spectrum of case scenarios.

The second premise is that distant kinship association will increase significantly hit rates, thereby substantially increasing investigative leads provided to law enforcement. Indeed, solving crime is a strong motivation and was a motivation to establish national DNA databases (even without a prior CBA). Thus, governments (and the people they serve) have made commitments to fight crime for justice, safety, and security reasons. Fundamentally, every hit obtained from a database search in the current government-maintained national database systems, either to a reference sample or to another crime sample, is due to recidivism as a hit currently relies on a previous entry in the database.

### Sexual assault cases

Beyond helping solve crimes, benefits of an effective and highly efficient DNA database lead development system, e.g., one based on FIGG, are that victims and families may gain some resolution and serial offenders may be identified earlier in their criminal careers which will prevent individuals from becoming future victims. The identification of perpetrators and reduction in victims will have substantial cost savings and benefits to the community, especially to victims and families, if investigative leads are acted upon. They also facilitate the criminal justice system while concomitantly bringing savings to taxpayers as well as reducing personal cost burdens.

### Proportion of DNA profiles in NDIS associated with sexual assault cases

Of the four case categories stated above, sexual assault cases are best suited for this CBA because there are more data collected regarding casework requests, sexual assault kit analyses, and DNA database uploads and hits. The Bureau of Justice Statistics [58] reported that for the USA in 2014 (most recent year data are available), there were 333,000 casework requests for biological analyses of which 45,000 were from sexual assault cases, which translates into ~ 13.5% of total requests are for sexual assault casework. The percentage of profiles from sexual assault evidence uploaded to NDIS may be equivalent to this percentage or higher today than 13.5% due to SAKI [55] providing resources and mandates to reduce sexual assault kit backlogs. Additionally, the percentage of profiles out of the total uploaded profiles may be higher for sexual assault cases because generally the samples may contain more DNA than some other crimes, such as property crimes. However, the number of sexual assault kits analyzed and reported in SAKI tends to support that 13.5% may be representative. Therefore, a percentage range of profiles comprising the total forensic profiles in NDIS from sexual assault cases was considered from an equivalent of 13.5% up to an arbitrarily higher proportion of 20%.

Over the lifetime of CODIS, there have been 587,773 hits ([57], latest data date October 2021), which translates to a highly successful ~ 51% of samples have yielded a hit. Assuming one hit per casework profile, which is not an entirely correct assumption because some counted hits are to more than one sample, there are 556,482 forensic profiles that have yet to hit to another profile. An assumption made is that the range of 13.5–20% proportion reflects the proportion of profiles from sexual assault cases in this no-hit category. This assumption may be inaccurate in part because the high serial recidivism hit rate of ~ 67% at NDIS (see below) observed with the SAKI data [55] may bias downward the hit rate in the no-hit category. Thus, there could be a lower proportion of serial recidivists associated with sexual assault cases in the no-hit category. Alternatively, the no-hit samples are merely from first offenses or serial offenders that have yet to be arrested or convicted of a crime that allows for a DNA profile to be uploaded to the database. Given that the FBI UCR [65] indicates that only 32.9% of rape cases were cleared by arrest or exceptional means, the bias assumption may not be large, if at all, and applying similar values for hit and no-hit categories was deemed reasonable. Thus, the number of samples related to sexual assault crimes in the no-hit category range from a low of 75,125 (i.e., 13.5% proportion of 556,482 no hit samples) to a high of 111,296 (i.e., 20% proportion of 556,482 no hit samples) (Supplementary Table 1).

### DNA profile upload rates

The current profile upload rate, based on SAKI data ([55], as of December 2022), is 41.6% (calculated as number of profiles uploaded divided by total completed kits = 33,398/80,325). An increase in the sheer number of markers [28] and sensitivity of detection would allow some samples that did not yield sufficient DNA profiles to cross the threshold and yield profiles that could be uploaded. However, such quantitative DNA data are not available. Given an overall greater sensitivity of detection and the large number of SNPs with the Kintelligence Kit (i.e., 10,230), an increase in the number of profiles uploaded can be expected. Therefore, an increase of profiles to meet upload requirements was arbitrarily estimated to be between 5 and 20%. Note that profiles that may be deemed insufficient for upload may still be useful profiles for investigative purposes, but they are not included in further analyses herein, although certainly they would yield investigative value and cost-benefits. Using the low and high estimates the increased upload over the lifetime of CODIS is between 57,213 (5% increase) to 228,851 samples (20% increase) which would be additional profiles available for developing investigative leads (Supplementary Table 1). Note that this estimate is based on 1,144,255 forensic samples currently in NDIS [57].

### Hit/investigative lead rates

Based on SAKI data, the current hit rate in CODIS is 47.3% (calculated as number of hits divided by total uploaded profiles = 15,784/33,398). Of these hits, 66.8% were to serial sex offenders and serial violent crime offenders (calculated as serial hit total divided by total number of hits = 10,550/15,784). FIGG offers the potential to increase the investigative lead rate substantially and thus can be especially effective for reducing the number of no-hit samples. Erlich et al. [67] reported that with GEDmatch profiles 76% of the kinship association cases (59 to 88% CI) shared > 100 cM and 10% shared > 300 cM (3 to 25% CI) which was similar to their simulated results with 1.28 million individuals. These authors further calculated that “with a database size of ~ 3 million U.S. individuals of European descent (2% of the adults of this population), more than 99% of the people of this ethnicity would have at least a single third-cousin match and more than 65% are expected to have at least one second-cousin match.” The hit ranges for FIGG herein are based on the GEDmatch data from Erlich et al. [67] and should be applicable for DNA data generated with the Kintelligence Kit. Snedecor et al. [26] have shown high sensitivity and specificity up to 4th order relatives (which is comparable to the degree of relationships considered by Erlich et al. [67]).

Applying these increased hit rates (59 to 88%) to the no hit samples in NDIS (*n* = 556,482) plus the predicted increase in uploaded samples (5–20%), there is an expectation that the number of additional investigative leads associated with sexual assault cases could be between 46,540 (59% hit rate and 13.5% proportion of profiles in NDIS) and 117,528 (88% hit rate and 20% proportion of profiles in NDIS) (Supplementary Table 1).

The increased upload and hit rates also can be used to predict annual performance. Using the estimate from Budowle et al. [49], 114,426 forensic profiles are uploaded per year (i.e., 1,144,255 forensic profiles divided by 10 years; October 2021 data). This estimate may not be entirely accurate as CODIS has been in existence for more than two decades; however, the upload rate has been much higher over the last decade or so. Applying the same increased hit rates (59 to 88%) and increased upload rates (5 to 20%) the number of investigative leads per year could range from a low value of 9570 (59% hit rate and 13.5% proportion of profiles in NDIS) and 24,167 (88% hit rate and 20% proportion of profiles in NDIS) (Supplementary Table 1). These numbers are greater than current expectations between 7307 (114,426 profiles per year, 47.3% hit rate, and 13.5% proportion of profiles in NDIS) and 10,825 (114,426 profiles per year, 47.3% hit rate, and 20% proportion of profiles in NDIS), respectively.

### Tangible and intangible costs

Tangible (i.e., medical, mental health, productivity, property loss, public services, adjudication and sanctioning, and perpetrator work loss) and intangible costs (i.e., quality of life) were obtained from Miller et al. [59] of which two of their categories applied to sexual assault and were listed as rape and other sexual assault. There were 354,779 crimes in these categories reported for the year 2017 of which ~ 38% were designated as rapes and ~ 62% were designated as sexual assault. Assuming this proportion holds for profiles uploaded to the government-maintained national DNA database, a weighted tangible cost would be $7419 and a weighted intangible cost would be $133,021 per case (derived from the data reported by Miller et al. [59]). It should be noted that society places high value on intangible benefits that particularly relate to solving crime, safety, and security; thus, those costs should not be ignored or considered less important than tangible costs (for example see [68]).

Cost savings based on the number of individuals that would not have become victims with an effective investigative lead tool can be estimated. Note that cost savings may occur for other cases not associated with serial recidivists but are not considered herein. The 66.8% serial offender hit rate is an indicator of the potential number of prevented victims due to early detection of serial offenders. While an earlier detection of recidivists would impact the reduction of victims in the hit category (calculated below), the no hit category would be more impacted with a process that generates more investigative leads. The current database structure relies on STR profiles and a direct hit in the database; if the source of the evidence has not been arrested or convicted previously, there will be no hit (or an unlikely adventitious hit). With a FIGG approach, a lead to identify the source of an evidence sample need not rely on a prior entry of the donor’s profile in the database. FIGG has a greater capacity to provide leads and to identify serial offenders earlier than the current database system. For this CBA, a reduction in future victims was set at an arbitrarily low 30% to a high of 67% (i.e., the current recidivist hit rate which may appear to be a high value but likely achievable given the high investigative lead rate of FIGG). Based on the range of increased investigative leads estimated above (46,540 to 117,528), the number of potentially prevented victims over the lifetime of CODIS could range from 13,962 (30% recidivism/victim reduction) to 78,744 (67% recidivism/victim reduction). Given this range of number of reduced victims, the tangible cost savings ($7,419 per case) from the no hit category could range from $103,583,611 to $584,201,944, respectively. Likewise, the intangible cost savings ($133,021) could range from $1,857,230,822 to $10,474,609,349, respectively (Supplementary Table 1).

With the current government-maintained national database system, the hit category, in theory, already generates investigative leads. However, to determine the benefit overall if FIGG were used routinely, the cost savings that would occur in the hit category also should be considered. For the hit category (*n* = 587,773), there potentially are between 14,747 (30% recidivism/victim reduction) and 83,173 (67% recidivism/victim reduction) victims that could have been prevented. The tangible cost savings ($7419 per case) from the hit category could range from $109,407,489 (30% reduction) to $617,056,878 (67% reduction). The intangible cost savings ($133,021) could range between $1,961,651,642 and $11,063,690,932, respectively. The total costs saved combining the hit and no-hit categories realized over the lifetime of CODIS could be between $212,991,099 to $1,201,258,822 (tangible) and from $3,818,882,463 to $21,538,300,280 (intangible) (Supplementary Table 1). The CBA supports that there are substantially more benefits in terms of costs, victim reduction, and quality of life that could have been realized if this technology had been implemented at the inception of forensic DNA databases.

For assessing annual performance expectations, an estimate of 114,426 profiles uploaded to NDIS per year was applied. Assuming similar values as used above, the benefits realized by the prevention of victims for tangible costs could be between $21,299,354 and $120,126,495 and for intangible cost savings could be between $381,892,614 and $2,153,841,008, respectively (Supplementary Table 1). The findings support that for annual estimates, there are substantial benefits to be obtained going forward using a FIGG approach.

It is unclear why there is such a high serial recidivist rate in the sexual assault DNA profiles in the hit category; an effective database system generating investigative leads should identify serial recidivists early on. Possible explanations include effects of the processing of the kit backlog and/or factors during the investigation and adjudication phases. Another potential factor could be that of the 80,325 completed kits reported in SAKI, only 23,426 had associated investigations (i.e., 29.2%). The number of cases associated with a CODIS hit and acted upon with follow-on investigative support cases is unknown. A highly functional investigative lead system is invaluable to fully use the data. To be fully effective, other parts of the investigative and legal systems (regardless of whether it is STR-based, SNP-based, or combinations thereof) need to be further assessed (beyond this CBA). Another factor could be that the hits are to other cases and not to reference profiles. For those hits, FIGG could be an improvement because the donor of the evidence does not need to be in the database. Lastly, more training may be needed for law enforcement personnel who may not be aware of the value of a database hit (generated by either for the current government-maintained national database or going forward the FIGG approach).

Initially, it was assumed that a DNA database would reduce the time law enforcement personnel dedicate to a case and in turn would allow them to investigate other cases, thereby enhancing efficiency. In contrast, data suggest that the labor by law enforcement approximately doubled for DNA hit cases compared with no-hit cases, possibly because law enforcement consider a hit as a high lead value to pursue. Therefore, for the simulation analyses, the costs of increased labor were considered at 50 h per case to accommodate potentially longer investigation times required for the genealogy work and investigative components of FIGG. The assessment assumes that this part of workload eventually will be transferred to law enforcement as such investigations fall under their purview. It also has the added benefit of reducing involvement of private citizens in the investigation phase. Some FIGG cases have taken much longer than 50 h. However, software tools have been and likely will be developed to facilitate tree building and selection (personal communication, S. Busch and S. Kramer at Indago.com) which in turn should reduce substantially the labor associated with the genealogy portion of the investigation.

### All other crimes combined

The same logic used for the sexual assault cases was applied to all the other violent and property crimes combined category (murder was treated separately) using the same increased upload rates and hit rates as above, while the proportion of profiles in NDIS, recidivism rates, and tangible and intangible costs applicable to this combined category of crimes were used. For simplicity of presentation, these categories (aggravated assault, other violent crime, burglary, larceny/theft, robbery, motor vehicle theft) were combined. A limitation is that little data exist on what proportion of profiles in CODIS can be attributed to each category. So, the proportions were assumed to be consistent with the proportion of crimes reported by Miller et al. [59] and a weighted average of the tangible and intangible costs across these crimes was used to mollify some of the effects due to unknown proportions. The proportion of these combined cases remaining after accounting for sexual assault cases is ~ 80 to ~ 86.5%. Murder, discussed separately below, is such a small proportion of the total cases and thus has little effect on these proportions. Roman et al. [61] found that 54.7% of the property crime cases studied yielded a profile that was uploaded to CODIS (higher than that of sexual assault cases), and these profiles resulted in a hit rate of 23.3% (lower than observed with sexual assault cases).

While these numbers are notable for developing investigative leads, only 17.2% of property crimes were cleared by arrest or exceptional means ([65], for year 2019). There is an expectation that a good portion of property crimes, assault, burglary, and robbery may be perpetrated by repeat offenders [69]. Therefore, the reduction in future victims was set at 30% (arbitrarily low) to 65% (the values based on data from Washington State [69]).

Over the lifetime of CODIS, the number of investigative leads generated in the no-hit category could have ranged from 275,792 (80% proportion of profiles in NDIS and 59% hit rate) to 508,313 (86.5% proportion of profiles in NDIS and 88% hit rate). Given a weighted tangible cost of $8,261 and intangible cost of $30,942 per case, the cost savings from the no hit category could range from $683,496,501 (80% proportion of profiles, 30% recidivism/victim reduction) to $2,729,462,461 (86.5% proportion of profiles, 65% recidivism/victim reduction). The intangible cost savings could range from $2,560,071,267 to $10,223,341,902, respectively. The tangible cost savings from the hit category could range from $693,352,469 to $2,513,546,523, respectively. Likewise, the intangible cost savings could range from $2,596,987,302 to $9,414,617,661, respectively. The total costs savings combining the hit and no-hit categories realized over the lifetime of CODIS could have been between $1,376,848,971 to $5,243,008,983 (tangible) and from $5,157,058,570 to $19,637,959,564 (intangible) (Supplementary Table 2). These results, although different quantitatively from the sexual assault data, also demonstrate that that substantially more benefits could have been obtained if the technology was in place from the inception of forensic DNA databases.

For annual performance expectations, a similar approach as used for sexual assault cases can be applied here. The number of victims reduced ranges from 17,103 to 67,939. The cost-benefits realized by the prevention of victims could be between $140,543,217 and $561,242,720 for tangible costs and between $526,411,842 and $2,102,163,449 for intangible costs, respectively (Supplementary Table 2). The same findings hold for the annual estimates in that there are substantial benefits to be obtained going forward using a FIGG approach.

### Murder

Although murder makes up a very small portion of all crimes reported, the costs can be quite high. The tangible and intangible costs are estimated to be $2,658,319 and $5,150,836, respectively, per case [59]. According to the FBI UCR [65], only 61.4% of murders and nonnegligent manslaughters are cleared by arrest or exceptional means. Thus, one could interpret from these data that there are 38.6% of murders that remain unsolved in a given year. For example, in the year 2017, 17,284 murders were reported [59]. Therefore, 6672 (0.386 × 17,284) murders remained unsolved in that year (Supplementary Table 3). Of those 6672 murders, it is unknown what portion yielded a DNA profile that was uploaded to NDIS and did not yield a hit. So, an arbitrary range of 30% (2001 profiles) to 50% (3336 profiles) was considered. This estimate may be low given the violent nature of murder and the potential for biological evidence being deposited at a crime scene. So, over a 10-year period and assuming similar values per year over that time period, there could be 20,015 to 33,358 profiles associated with murder cases in the no-hit sample category (Supplementary Table 3). Martin et al. [64] reported that there are more than 100,000 unresolved homicides over the past 20 years, which is comparable to the 20,015 to 33,358 numbers over ten years estimated herein. Applying the same increased uploaded profiles and hit rates as above, over the 10-year period alone the number of investigative leads that could have been obtained from the no-hit category could range from 12,399 (30% uploaded profiles and 59% hit rate) to 35,226 (50% uploaded profiles and 88% hit rate) (Supplementary Table 3).

Murder is one of the most serious of all crimes resulting in loss of life, affecting personal and community safety, creating undue stress and mental health issues, loss of productivity, impacting property value, and at times creating public panic. Solving murders as quickly as possible has a huge impact on intangible costs, i.e., quality of life. Thus, the total intangible cost savings are substantial and likely difficult to fully quantify if a murder is solved expeditiously. However, the tangible and intangible costs associated with these additional hits are in the billions to trillions of dollars over the ten-year period (data not shown, but extrapolated from [62–64]), which are quite substantial and a huge burden to society.

Solving murders with the assistance of FIGG may not generate savings as is anticipated for sexual assault and all other crimes categories, except possibly for reducing investigative costs and potentially reducing some intangible costs. While more investigative leads can be generated which help solve more cases (an important and obligatory goal), they do not necessarily result in a reduction of murder victims. However, for cases involving serial murderers, savings may occur. Serial murder is defined as “The unlawful killing of two or more victims by the same offender(s), in separate events” [70]. While names like Ted Bundy, Gary Ridgway, and William Gacy Jr are well known and may seem to infer that serial murderers are mostly from the relatively distant past (a generation or two ago), serial murderers are present in the twenty-first century and continue to commit crimes, and some cases remain unsolved, such as the Zodiac Killer and the Long Island Serial Killer. Although serial murder is relatively rare per se, the number of victims can be substantial. Supplementary Table 4 provides a non-exhaustive list of known US serial killers since the mid-twentieth century [62, 70–75]. The number of victims (> 1000) listed is based primarily on convictions and confessions, and the overall number could be much higher. Quinet [62], Pappas [63], and Martin et al. [64] suggest that the number of serial murderers is higher with estimates of 2000 serial killers who have never been prosecuted, up to 15% of murders may be due to serial killers, and between the years of 2010–2015, there was an average of 54 serial killers per year. The number of unaccounted serial murder victims ranges from a low of 182 to a high of 1832 per year [62].

As can be seen from the numbers (some of which are listed in Supplementary Table 4), multiple people were murdered before the cases were solved. Some delays in solving cases may be due to a lack of coordinated efforts, whether victims have low-risk or high-risk lifestyles, lack of forensic evidence, lack of technology, and lack of resources. Yet, the first indications of a serial murder typically arise through forensic and behavioral evidence [70]. One tool to identify these individuals early in their criminal careers is DNA analysis. However, with the current government-maintained national database system (e.g., NDIS), the individual needs to be in the database to develop an investigative lead regarding the source of the forensic sample(s) (although cases can be linked by searching profiles within the forensic profiles index). With FIGG, investigative leads can be developed more readily in a similar manner as was done in the Golden State Killer case (whose profile was not in the local, state or national databases) [29].

Assuming 15% of murders [64] may be associated with serial killers, perhaps as many as 15% of murders may have been prevented with more and earlier investigative leads, and to be conservative, perhaps as few as 5% may have been prevented. Thus, the number of reduced victims could have ranged from 620 to 5284. The tangible and intangible cost savings could have been from $1,648,053,202 and $14,046,361,433 (5% recidivism/victim reduction and 59% hit rate), respectively, to $3,193,315,687 and $27,216,637,334 (15% recidivism/victim reduction and 88% hit rate), respectively. Annually, the number of reduced victims would range from 62 to 528. The savings could range from $164,805,320 to $1,404,636,143 (tangible) and $319,331,569 to $2,721,663,733 (intangible), respectively. The analyses indicate that for murder, which tends to be the mostly costly crime on a per crime basis, lives could be saved and substantial cost savings can be obtained by using a FIGG approach.

Table 5 combines the savings for all crime categories over the equivalent of the lifetime of CODIS and what would be projected annually. The savings are quite substantial.

**Table 5:** Total projected cost savings over lifetime of CODIS and annually

	Tangible costs	Intangible Costs	Total
Lifetime savings			
Sexual assault cases			
Lowest cost saving	$212,991,099	$3,818,882,463	$4,031,873,562
Highest cost saving	$1,201,258,822	$21,538,300,280	$22,739,559,102
Other crimes			
Lowest cost saving	$1,376,848,971	$5,157,058,570	$6,533,907,540
Highest cost saving	$5,243,008,983	$19,637,959,564	$24,880,968,547
Murder			
Lowest cost saving	$1,648,053,202	$3,193,315,687	$4,841,368,889
Highest cost saving	$14,046,361,433	$27,216,637,334	$41,262,998,767
Total			
Lowest cost saving	$3,237,893,272	$12,169,256,720	$15,407,149,992
Highest cost saving	$20,490,629,239	$68,392,897,177	$88,883,526,416
Annual savings			
Sexual assault cases			
Lowest cost saving	$21,299,354	$381,892,614	$403,191,967
Highest cost saving	$120,126,495	$2,153,841,008	$2,273,967,503
Other crimes			
Lowest cost saving	$140,543,217	$526,411,842	$666,955,060
Highest cost saving	$561,242,720	$2,102,163,449	$2,663,406,170
Murder			
Lowest cost saving	$164,805,320	$319,331,569	$484,136,889
Highest cost saving	$1,404,636,143	$2,721,663,733	$4,126,299,877
Total			
Lowest cost saving	$326,647,891	$1,227,636,025	$1,554,283,916
Highest cost saving	$2,086,005,358	$6,977,668,191	$9,063,673,549

### Monte Carlo simulation of key criteria for annual cost-benefits

So far, the costs and benefits have been estimated as low and high values. While the benefits appear to be large, the aforementioned analyses were based on a static model (i.e., selected single values) and do not consider the full range of possible outcomes. Although some data for the analyses were readily available, the forensic community does not necessarily collect or report performance data that could reduce uncertainty in some estimates. To generate best estimates that reflect the uncertainty with the extant data, a Monte Carlo simulation and sensitivity CBA was performed on the potential benefit of the application of a large SNP panel and increased investigative lead rates associated with FIGG. This approach allows multiple inputs over realistic value ranges and samples the range spaces (see Tables 2, 3, and 4) simultaneously to generate best estimate summary statistics to obtain probabilistic outcomes so that the behavior of real-life systems can be approximated better. The key outcomes were the number of victims that may be reduced and tangible and intangible costs obtained.

Supplementary Figs. 1–4 display the probability distributions for the three key outcomes for sexual assault, all other crimes, murder, and all three categories combined, respectively and Supplementary Figs. 5–8 display the accompanying sensitivity analyses. While the ranges are similar to those of the static analyses above, the mean values (and standard deviations) are considered best estimates for assessing the impact benefits. The number of reduced victims per year are, on average, for sexual assault, all other crimes, and murder is estimated to be 8700, 42,162, and 258. The simulation of all crimes combined estimated that on average 51,120 victims could be reduced each year with this technology. Average tangible cost-benefits ranged from $65,113,075 (sexual assault) to $659,066,513 (murder) with a combined cost savings of $1,135,559,428 per year. Average intangible costs ranged from $1,154,211,092 (sexual assault) to 1,304,828,280 (murder) with a combined cost savings of $3,738,315,373 per year. There are, on average, over 50,000 people per year that may not become victims and total savings per year are around $4.8 billion, if FIGG were to be employed. The numbers are quite telling.

### Missing person cases

The sheer volume of missing and unidentified person cases worldwide is a humanitarian tragedy to magnitude families and communities and poses major challenges to agencies responsible for identifications. In the USA alone, 4400 new unidentified decedents are recovered across the country each year, with approximately 1000 of these cases remaining unidentified and becoming cold or unresolved cases [76]. Identifications are difficult due to the quantity and quality of remains, the lack of adequate family reference samples, and lack of sufficient resources to support forensic analyses. From 2003 to 2022, the Center for Human Identification generated 19,760 family reference sample profiles, 8248 unidentified remains profiles and 421 missing persons profiles, and there were 3569 associations reported to submitting agencies (unpublished data). Thus, over the lifetime of entering missing persons data into the current government-maintained national DNA database, the association rate has been ~ 43.3%. On qualitative and semi-quantitative levels, FIGG approaches can increase the success of identifying remains as they are more sensitive than current methods, are not dependent on family reference samples of a particular lineage (although mitochondrial DNA is successfully typed in the majority of human remains samples (unpublished data), and Y chromosome data can be informative for FIGG [77]), and may be able to assist in identifying individuals with more distant relatives than are currently relied on (typically first degree relatives).

While it is obvious that there are tangible and intangible costs associated with losing family members, to the best of the authors’ knowledge, there do not seem to be data associated with this aspect of forensic identifications. Moreover, identifying victims, either single individual cases or as a result of mass disasters, in itself does not prevent future victims per se. The motivation to identify the missing is primarily humanitarian, although a portion of decedents may have met their death due to a criminal act. Therefore, for this CBA, an assessment is whether the costs of performing large SNP analysis would be reasonable considering the benefits of a higher performing technology. Generally, STR typing by CE offers a poorer sensitivity of detection and a lower kinship resolution compared with the potential of NGS. Additionally, Ge and Budowle [78] estimated for parent–child and full-sibling cases false negative rates can be 1 in 770 and 1 in 160, respectively, with an STR kit, like Globalfiler (Thermo Fisher Scientific). More distant relationships would have higher false negative (and false positive) rates, assuming a binary threshold approach. False negatives are particularly vexing in full-sibling cases in which the true relationship is half-siblings. The panels of STR markers simply do not have sufficient power to correctly associate relationships beyond first degree. However, the dense and large SNP approaches can substantially reduce the false positive and false negative rates to meaningless levels for typical first degree reference comparisons with missing persons cases, as well as enable more distant relationship reference samples to be used effectively in missing persons and disaster victim identifications. Moreover, Snedecor et al. [26] showed that even with a reduced call rate (40–80%), kinship up to 3rd degree relatives was obtainable. Thus, partial profiles still can provide strong information for the types of missing persons cases and pedigrees traditionally investigated and improve upon the 43.3% association rate. SNP typing is likely to yield a higher call rate than STRs, simply because the DNA fragment sizes required for typing are shorter (for example see [8]) and with the Kintelligence Kit 10,001 of the 10,230 SNPs reside in amplicons < 150 bp [28]. These features alone, and less reliance on lineage associations, should support the use of FIGG in humanitarian efforts to identify missing persons and human remains.

The costs to generate DNA profiles with FIGG are comparable to those associated with STR and mitochondrial DNA typing. For example, for the years 2020–2021, the reagent costs to generate profiles (autosomal and Y STRs and mitochondrial DNA) at the Center for Human Identification for a reference sample were ~ $100 and for a human remains, sample were ~ $100-$370 (cost dependent on the quality of the remains) (unpublished data). The cost of a large SNP profile generated with the Kintelligence Kit could be projected to be ~ $290 and ~ $365 (Supplementary Table 5) for a reference and human remains samples, respectively. Given the added advantages, the increase in reference sample cost is comparable and more than justified. Also note that typing one set of markers instead of potentially three sets of markers (autosomal STRs, Y STRs, and mitochondrial DNA) would reduce the difference in costs (due to labor reduction) between current analyses and large SNP analyses. When added to increased sensitivity of detection, analysis on degraded DNA, and distant kinship associations, the marginally greater costs are well justified.

### Basic costs to build a large SNP-based laboratory and database system

There are qualitative and quantitative benefits with employing a large SNP panel and FIGG which include increasing the number of typable samples, generating more database investigative leads, supporting more investigations, faster case resolution, and prevention of future crimes with substantial tangible and intangible savings. The above data were generated assuming a mature laboratory and database system were in place. Obviously, such a system is nascent at best. Given these benefits, the basic investment costs for building and operating a laboratory and functional database system need to be determined. Those aspects that would be the same whether it is a STR-based system or SNP-based system are not considered as that cost would be borne either way. For example, the costs of sample preparation, labor for generating a profile, personnel, and maintaining a database are not added as those costs would be approximately the same for NGS and CE approaches. The basic costs to build the system would be reagents/consumables, sequencing instrumentation (and supporting software), robots for library preparation, validation studies, and training. This part of the analysis also is based on using the Kintelligence Kit (with various per sample projected costs) and either the MiSeq FGx system or a NextSeq 2000 system (Illumina). Since technology change and implementation take time, the costs are distributed over ~ 10 years. Annual costs are provided, where appropriate, as well.

### Reagent costs

The cost of generating a large SNP profile on a per sample basis will vary depending on the number of samples per sequencing run and the cost of a library preparation kit as well as the throughput needs of a laboratory. This part of the exercise is bit of a “chicken and egg” issue as high-volume kits are not available and likely would not be available until demand for high throughput sample processing is needed (as it currently is for CE). The estimated sample costs using the Kintelligence Kit and accompanying sequencing platforms are provided in Supplementary Table 5. The cost of a current Kintelligence Kit for generating 12 libraries is $11,499, and the MiSeq FGx Reagent Standard Kit for sequencing is $1500 (these prices may vary due to purchasing agreements with the vendor). If three samples were sequenced per run on a MiSeq, reagent cost would be $1458.25 per sample. However, Antunes et al. [79] have shown that sample plexity could be increased with little loss in the SNP call rate. Therefore, for reference samples the cost could be reduced to $1,008.25 for 30 libraries per sequence run and $1,083.25 for 12 forensic sample libraries. For forensic samples, the plexity per sequence run may be reduced to achieve higher read depth and thus sample cost would increase slightly. For example, if only six forensic sample libraries were run, the per sample cost would be $1208.25.

It is reasonable to assume that the cost per sample likely will be lower with a high-volume sample kit. Assume that a 96 sample Kintelligence Kit was produced, and the cost was approximately $23,000, i.e., ~ double the cost of the 12-sample kit. Under this scenario, if three samples were sequenced on a MiSeq (including the accompanying Standard Reagent Kit), sample cost would be $739.58 per sample. For 30 reference samples, the cost could be reduced to $289.58 per sample. Similarly, for 12 casework samples, the cost would be $364.58 per sample. If the library preparation kit per sample cost was further reduced to $120.00, then the cost with three samples sequenced on a MiSeq (including the accompanying Standard Reagent Kit) would result in a per sample cost of $620.00. For 30 reference samples, the cost could be reduced to $170.00 per sample. Similarly, for 12 casework samples, the cost would be $245.00 per sample. These costs are about 5–10 × greater than that of CE generated STR profiles.

If a NextSeq 2000 instrument was used, a P2 flow cell (with about 400 million reads) would cost $2,737. This sequencing system would allow for more than a tenfold increase in sample throughput (assuming sufficient unique DNA identifiers are available) and would be well-suited for database laboratories. Sequencing 96 or 300 libraries per run (at $239.58 library preparation/sample) would result in a per reference or forensic sample cost of $268.09 and $248.70, respectively. If library preparation per sample costs $120.00, then the cost to generate a profile using the NextSeq 2000 could be $148.51 and $129.12, respectively (Supplementary Table 5). These lower costs are speculative, but there is an expectation of sample costs lowering with advances in technology and higher volume manufacturing if or when there is more demand. An advantage of sequencing technology is that future technology developments likely will be backwards compatible.

### Caseload reagent costs

Caseloads are different per laboratory, and thus, budgets will vary. Therefore, a national level cost was entertained to provide insight into investment costs. Using the casework requests of 330,000 per year [58] and assuming 5 samples per request [49], there would be about 1,665,000 samples analyzed annually in the USA. This number of requests may be low, so one can also consider 400,000 requests with 5 samples per case for a total of approximately 2,000,000 samples analyzed annually. Assuming a sample cost of $289.58, the total cost would be between $477,807,000 and $579,160,000 per year and with a more optimistic $170.00 per sample the total cost would be between $280,500,000 and $340,000,000 per year (Supplementary Table 5). Other permutations could be considered that would vary these costs slightly. Assuming the current cost per sample for STR profiles developed by CE is approximately $40, then the current costs would be between $66,000,000 and $80,000,000 per year. Therefore, an increase in reagent cost nationally for the generation of large SNP profiles would be ~ 4–10 × current costs per year.

Erlich et al. [67] showed that a reference database of ~ 3,000,000 people per population would be sufficient for 99% of searches to yield at least one third-cousin association. Building a reference database of, for example, 10,000,000 individuals would take time. But using a 10-year period and 1,000,000 samples per year, the cost at $289.58 or $120/per sample would be between $289,580,000 and $120,000,000, respectively, per year. Of course, this cost could be reduced by relying in part on current voluntary profile databases.

### Instrumentation costs

To date, 163 labs in the USA already have a MiSeq (personal communication Swathi Kumar, Verogen). Assuming a cost of $180,000 per sequencing system (to include instrument, warranty, Universal Analysis Software, and service support for the first year), then purchasing an additional 200 instrument systems would be $36,200,000. For database laboratories and casework laboratories that desire higher throughput a NextSeq 2000 instrument may be better suited. At $373,000 per NextSeq 2000 system the cost for 100 instruments would be $37,300,000. The combined total initial outlay for sequencing instrumentation would be $73,500,000 or $7,350,000 per year (Supplementary Table 5).

Robots would be needed to facilitate sample preparation, particularly for generating libraries. The cost of two robots per laboratory (200 laboratories) at $150,000 per robot would be $60,000,000 or $6,000,000 per year (Supplementary Table 5).

### Miscellaneous costs

Other costs would include validation studies. Assuming it would cost $100,000 each for 200 labs to perform a validation study, the cost would be $20,000,000 at a per year cost of $2,000,000. With sharing of data among laboratories validation costs likely will be lower. As there will be other costs not captured with the basics (such as servers, data interpretation software, data storage, LIMS modifications, and some additional labor), another $50,000,000 per year was added to the build and operational costs.

Projected costs per year are shown in Table 6 (and Supplementary Table 6) and would range from $525,350,000 and $943,090,000. The average savings per year total is more than $4.8 billion. Thus, these costs to build a functional laboratory and database system are well justified. It also should be stressed again that a technology that reduces the number or prevents victims from ever becoming victims — on average more than 50,000 projected per year — should be sufficient motivation to acquire and implement such technology. The CBA supports that FIGG is a solid investment.

**Table 6:** Projec﻿ted additional costs per year for casework and database work, instrumentation, and other operational demands

	$289.58/sample	$120/sample
Casework	$579,160,000	$340,000,000
Database work	$298,580,000	$120,000,000
Sequencers	$7,350,000	$7,350,000
Robots	$6,000,000	$6,000,000
Validation studies	$2,000,000	$2,000,000
Miscellaneous	$50,000,000	$50,000,000
Total cost/year	$943,090,000	$525,350,000

### Cost per hit/investigative lead

For the last 19 years, the Debbie Smith Act has provided ~ $151 million/year. Thus, this act alone has funded $2,869,000,000 to support forensic DNA typing and related activities, such as backlog reduction. According to the Congressional Research Service, “The bulk of the programs focus on providing state and local governments with funding to reduce the backlog of forensic and offender samples waiting to be processed and entered into the NDIS” [80]. Using these data, a general cost per sample hit in CODIS can be calculated. A range around the funding from the Debbie Smith Act of $2,000,000,000 to $3,000,000,000 was applied to account for federal funding support, although the actual proportion of the bulk of funding is not readily known. This estimate is likely low, but reasonable, as it does not include state contributions to their operations and database support or the cost to the FBI to manage, operate, and continuously improve the database functionality. Given 587,773 hits reported to date at NDIS, the cost per hit in CODIS ranges from $3403 to $5104 per hit. Table 7 (and Supplementary Table 7) displays the projected cost/investigative lead. The range for the extreme costs/investigative lead can be $4,348 to $13,304. The costs eventually should be lower once the laboratory and database system has reached a mature level.

**Table 7:** Projected cost per investigative lead with the large SNP panel approach

Sample/year uploaded	Increased upload rate	No. of samples/year	Investigative lead rate	Investigative leads/year	Cost per year	Cost/investigative lead
114,426	5%	120,147	0.59	70,887	$943,090,000	$13,304
114,426	20%	137,311	0.88	120,834	$525,350,000	$4,348

These estimates are modest. As the database reaches an optimum number of samples representing an association rate, in theory, can reach 99%. Additionally, the costs herein do not entertain another technology that will reduce sequencing costs. Already there are companies that suggest that their technologies can bring sequencing costs down to ~ $100/human genome [81–83]. Therefore, it is conceivable that sequencing platforms will be able to analyze samples at similar costs to those of CE, but with higher resolution and increased sensitivity of detection in the foreseeable future.

### Microarrays

Up to this point, the CBA considered solely using a NGS approach to generate data. However, microarrays have been used almost exclusively by the direct-to-consumer companies to populate their databases, and the data in, for example, GEDmatch and FamilyTree DNA are overwhelmingly from microarrays. The kinship SNPs in the Kintelligence Kit are contained within Illumina Infinium CytoSNP-850 K BeadChip, Infinium Global Screening Array, and others. Thus, reference databases may be able to grow at a lower cost than projected with the Kintelligence Kit or WGS. The Illumina Infinium CytoSNP-850 K BeadChip can process 8 samples simultaneously, and kits can be purchased to analyze 8, 16, 48, and 96 samples [84]. The price for an 8-sample kit is $2040.00 and for a 96-sample kit is $24,480.00, which is $255/sample. The Infinium Global Screening Array can process 24 samples simultaneously, and kits can be purchased to analyze 48, 288, and 1152 samples [85]. The price for a 48-sample kit is $2352.00 and for a 1152-sample kit is $56,448.00, which is $49/sample. So, using the same 10-year period and 1,000,000 samples per year as above the cost (at $49, the lower cost per sample for the two microarrays), the database work reagent cost would be $49,000,000 per year for populating reference databases. Thus, the annual cost would reduce to a cost of $454,350,000 to $693,510,000, and the cost per investigative lead would be between $3760 and $9783 (Supplementary Table 7), which are comparable to current CE-based hit rate costs.

The limitation, however, with the use of microarrays, in addition to requiring a large amount of input DNA, is that they contain potential genetic data with reasonable positive predictive power that could expose personal, privacy information about an individual. Budowle et al., [86] and Marshall et al. [87] recommended (for whole mitochondrial genome sequence data) those SNPs that may provide such information could be filtered so only SNPs with little or no predictive power are reported. However, the raw data would still contain the SNPs which some jurisdictions and stakeholders might consider a risk to privacy. Those limitations and benefits need to be weighed [46]; nonetheless, this CBA considered only the costs.

### Limitations

Generally, one could assert that this CBA is not comprehensive and does not capture all costs. The assumptions were based on extant data and personal experience, but data are limited. If more quantifiable data were available, the costs and cost-benefits could be better estimated. However, the overall outcome of a substantial cost–benefit would still be supported. Indeed, an average total cost–benefit of greater than $4.8 billion per year is a 5 to 10 times return on the investment to build the laboratory infrastructure and populate a database system. Even if additional operating costs were doubled, there would still be a substantial return on the investment. Additional limitations of this study are:

- Some assumptions may overestimate, and some may underestimate the costs and benefits. Most of the parameter ranges or values may seem reasonable with some being relatively low-end estimates for the ranges, others on the upper end could be considered optimistic. The cost for FIGG may be greater or lower; costs were based on high volume kits with lower library preparation costs, high throughput sequencing, and notably higher investigative lead rates. The most optimistic estimates may be the costs for consumables to generate a large SNP panel profile with the Kintelligence Kit. There is a reasonable expectation that costs will decrease over time but to what degree is unknown. Regardless, if the lowered prices used for the CBA were too optimistic, they could serve as indicators of what consumable expenses should be to make the cost–benefit worthwhile. However, even if the price of consumables remains higher than what was used for this CBA, the projected benefits are notably higher than the estimated costs than the infrastructure investment; so, there is room for increased cost and yet still being able to garner cost savings (tangible and intangible).
- The estimates for potential upload increases could be improved if laboratories collected data on amounts of DNA recovered and portion of profiles generated that could not be uploaded because of current policies and technology limitations. The portion of samples that yielded some STR data but just not enough to meet current upload criteria may be the best candidates to yield more useful data with a large SNP panel.
- This CBA focused primarily on cost savings due to a reduction in recidivism crime. Thus, the cost savings are likely underestimates of the value of FIGG as there are cost-benefits for assisting in solving crimes that are not attributed to serial recidivists. Additionally, those profiles that do not meet upload requirements may still be useful and these samples were not addressed herein but would contribute to operational benefits given the greater versatility of a large SNP panel.
- The proportion of samples associated with each crime may not be accurate as they were based on the proportion of reported crimes. There may be differences in typing success and upload rates that may affect these values. The average tangible and intangible costs from Miller et al. [59] were used in this CBA but may not reflect the proportions in the actual database. However, averaging, the ranges used, and the simulation data may moderate some of the potential inaccuracies (particularly with the simulation data).
- The analyses did not include the costs that GEDmatch and FamilyTree DNA charge per upload/search. Clearly, those costs would increase expenditures. However, this CBA assumed a mature large SNP database system and thus, such costs would not be incurred routinely. Alternatively, the continued use and growth of such privately owned databases likely would reduce the cost of populating the databases. To date, for example, GEDmatch is populated with profiles from people who voluntarily have uploaded their data. If this trend were to continue (say for the next 10 years), the database could grow substantially at no cost to the government. The costs estimated herein to populate a database might be substantially higher than the cost of searching a privately owned database. Thus, not including a search cost should not impact the estimates and outcomes.
- The data used are US centric, as the information was more readily accessible for this country. The costs and benefits may not be generalizable beyond the USA. The concepts or model, however, should apply, and interested parties could substitute their estimates to carry out a CBA.
- It should be realized that not all associations made with large SNP profile and kinship searches will translate to an effective end result of identification (which also is a concern with the current government-maintained national database and criminal justice systems). Specifically, for FIGG, there are limitations with public records and genealogy surveying. These limitations may not be readily resolvable if records do not accurately reflect relationships as well as the performance (i.e., capabilities/expertise) of genealogists is unknown. However, if a search yields multiple candidate relatives, an inaccurate record with one potential lead may be overcome by others, although it may create more work for investigators. These limitations require more research to assess downstream practices of FIGG.
- From the SAKI data [55], not all cases are acted upon, which could reduce the benefits of any investigative lead generated based on either the current STR approach or a FIGG one. Data are needed to determine the reasons for why such cases are not pursued further and what portion of non-acted upon cases have DNA results. Additionally, training and other infrastructure developments may be needed to increase the number of cases acted upon for assessing the value of FIGG, but also just as importantly for the current government-maintained national database system.
- The SNPs in the Kintelligence panel were searched against ClinVar to reduce the risk to medical/health privacy. With these many SNPs, there still may be some that have associations that may be detected especially with advanced computational capabilities. Associations though do not necessarily translate to positive predictive power, especially with many diseases being multigenic. However, to continue with the same intent of reducing privacy risks, the SNPs should be reviewed on a regular basis if new knowledge may indicate that any specific SNPs may impact genetic privacy.
- The investigative lead rates for FIGG used in this study ranged from 59 to 88%. One might assert that the upper end of this range may be too optimistic. Recently, Whitman [88] reported that 92% of FIGG cases worked by the FBI were solved in 12 months. These data support that the upper end rate may be reasonable, but it is unknown if there were selection criteria for these cases that may have impacted the success rate. Selection criteria and success rates should be collected and reported to determine what may be reasonable expectations of association rates with FIGG.
- With STR typing and a minimum number of loci required to upload to a database, a partial profile can be problematic. A 10–40% STR profile may not be sufficient for upload. However, a similar percentage of SNPs (i.e., 1000–4000) could be quite informative, especially for one-to-one comparisons and first-degree relative associations. The greater information and versatility across a wider range of partial profiles with a large SNP approach was not factored into the CBA, but clearly is another benefit.
- There are likely costs that could have been included. To compensate $50,000,000/year were added. Given the overall benefits, this value could be increased several fold and benefits would still be gained.
- Mixture evidence was not addressed herein, primarily because these analyses were based on the use of databases to develop investigative leads. Most entries into for example CODIS are single source or readily resolvable profiles, and often the major contributor profile of a mixture. These samples mostly would be considered simple mixtures. Thus, such mixture evidence would be entertained whether a STR/CE or NGS approach or a large SNP panel approach was used. Currently, because of the greater number of alleles per locus compared with a SNP locus, STRs would appear to be better suited for complex mixture deconvolution. However, mixture deconvolution of complex SNP mixtures is not a new concept. Indeed, more than a decade ago Homer et al. [89] proposed a methodology to identify contributors of a mixture (simple, complex, abundant, and trace) from dense SNP microarray data. The trace levels suggested by Homer et al. [89] are below those achieved with STR analyses. More recent studies [90, 91] have reported that probabilistic SNP genotyping of low DNA concentrations is feasible. Likely software will be developed in the near future from several sources to facilitate mixture interpretation. It seems reasonable to expect that a large SNP panel may be suitable for analysis of complex mixtures, and future studies should elucidate to what degree deconvolution is attainable.
- Lastly, the reference profiles in CODIS in effect depreciate over time. As convicted offenders age the likelihood that they will continue to commit particularly violent crimes reduces. Thus, the investigative lead value for profiles from aged individuals that have been in the government-maintained national DNA databases for substantial time are less valuable from the perspective of generating leads for an active crime. In contrast, with a FIGG approach, which is based on kinship association, lead value of profiles can extend for multiple generations. The depreciation costs of profiles in the government-maintained national DNA databases versus the FIGG approach were not considered but add to a cost–benefit with SNP-based approaches. Data on the hits in, for example CODIS, and ages of associated individuals per crime would allow for an assessment of depreciation.

## Conclusions

This CBA assessed the potential benefits that could be gained by using a FIGG approach in lieu of the current STR approach. This study shows that a cost of less than $1 billion per year (over the next ten years) can reap, on average, > $4.8 billion in tangible and intangible cost-benefits per year. Thus, while the increase in the laboratory budget may seem notable, compared to its current budget, the benefit to society is immense making the laboratory investment a nominal cost. With these savings, some latitude in assumptions can be tolerated, and the overall conclusions in this CBA still would be well supported. More importantly, there should be investments in an infrastructure that have a direct impact on quality of life. The key outcomes support that FIGG could yield substantial tangible and intangible cost savings and more importantly prevent the number of individuals that would become victims (on average > 50,000 per year). The savings are far greater than the investments needed for the laboratory and database systems and thus support moving forward with FIGG from a cost–benefit and victim reduction perspective.

As previously reported [49], the costs to society due to crime are likely low estimates because the number of cases reported is low [60, 92, 93]. For example, it is estimated that only 5–25% of victims report sexual assault. Likely, a portion of the non-reported cases are committed by serial offenders. In such cases, victim reduction and substantial cost-benefits also would occur. Thus, the estimates herein are underestimates of the potential benefits obtained. Furthermore, increased success with DNA typing and more hits being investigated may generate more confidence for victims to report their assaults as well as provide additional safety, security and resolution.

Higher hit rates also may impact wrongful convictions and wrongful arrests as searching the FIGG database could produce leads to other more likely suspects. As previously pointed out [49], tangible and intangible costs of wrongful convictions and wrongful arrests on a per case basis may be commensurate or greater than those of rape and murder, especially when considering the social stigma, loss of freedom, loss of productivity, pain and suffering, government liabilities, and subsequent settlements.

As stated above, the data herein are US centric primarily because the data were readily accessible for this CBA. The specific cost-benefits may not readily generalize to other countries as the US population size is different, the pursuit of perpetrators of crime may vary, the costs to society may differ, etc. Thus, the total cost-benefits may not be as impressive for some other countries. However, the potential benefits could be determined in a similar fashion as was performed in this CBA and likely still would be quite notable relative to the particular country.

Although this CBA considered FIGG solely, it does not mean that the current database system needs to be wholly replaced. Indeed, a better approach may be to incorporate FIGG into the current infrastructure so that the benefits of both systems may be leveraged to generate investigative leads as effectively as possible. To refine CBAs for FIGG and for that matter any technology or service, laboratories and criminal justice systems should start capturing performance data (for casework operations and database operations) in more detail to support better relevant assumptions. There is a need to collect data on the number and range of DNA/amount of profiles generated. Laboratories should consider collecting metrics that assess the performance of their analytical systems. Laboratories also could capture some of these data when performing validation studies to determine effectiveness. With such data effective CBAs can be generated, and laboratories can achieve their goals of providing the best services to support the criminal justice system and more so that society can better assess the benefits that may arise.

## Supplementary Information

Below is the link to the electronic supplementary material.

Supplementary file1 (DOCX 680 KB)

Supplementary file2 (XLSX 28.8 KB)
