# Supplementary material for: A cost–benefit analysis for use of large SNP panels and high throughput typing for forensic investigative genetic genealogy
Source: Int J Legal Med. 2023 Jun 21;137(5):1595–614. doi: 10.1007/s00414-023-03029-7 (PMC10421786; doi:10.1007/s00414-023-03029-7)
Supplement: Supplementary file 1 — Supplementary file1 (DOCX 680 KB) [file 414_2023_3029_MOESM1_ESM.docx]

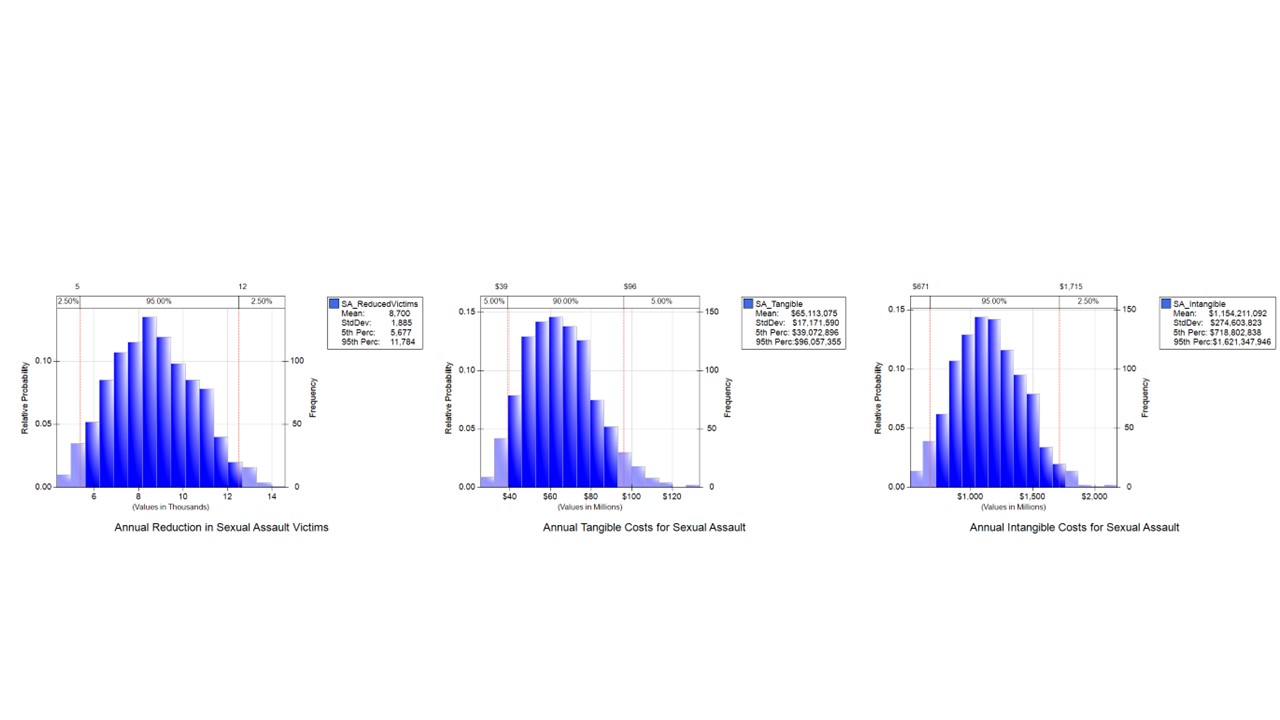


Supplementary Figure 1. Monte Carlo simulation for key outcomes of reduction in victims (left histogram), tangible cost savings (center histogram), and intangible cost savings (right histogram) for sexual assault cases per year. Accompanying sensitivity analyses are shown in Supplementary Figure 5.


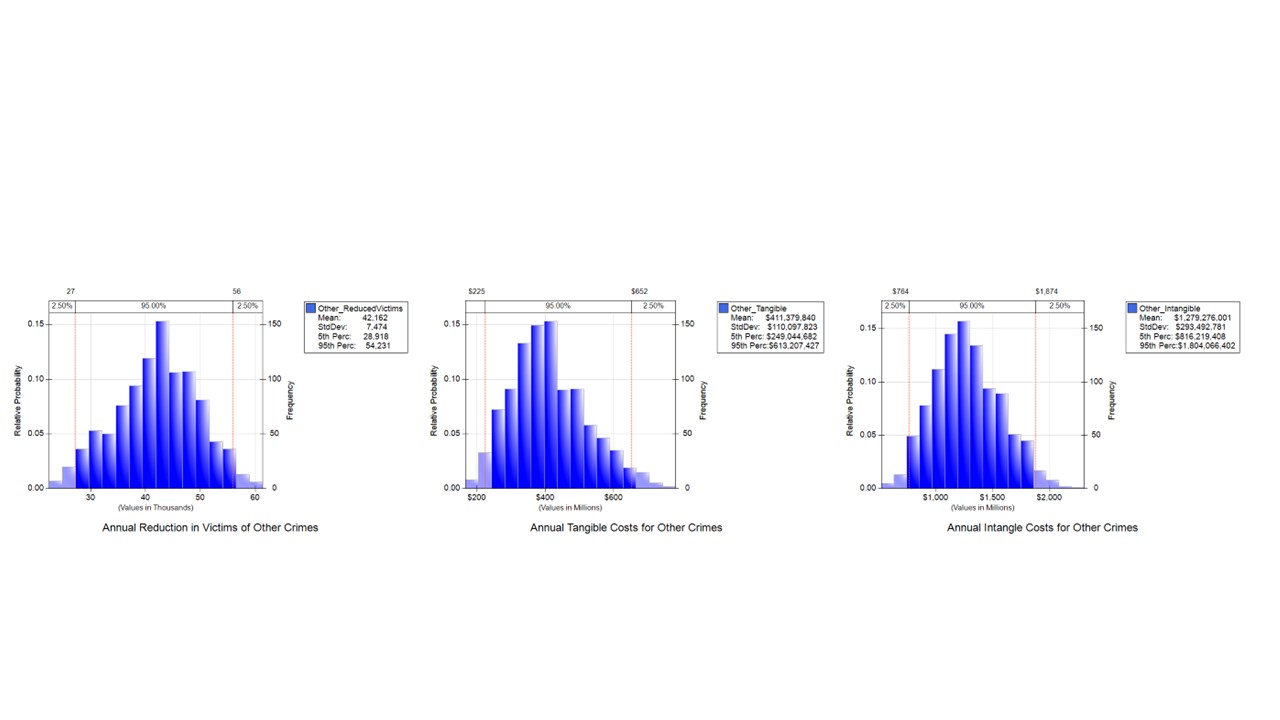


Supplementary Figure 2. Monte Carlo simulation for key outcomes of reduction in victims (left histogram), tangible cost savings (center histogram), and intangible cost savings (right histogram) for other crime cases per year. Accompanying sensitivity analyses are shown in Supplementary Figure 6.


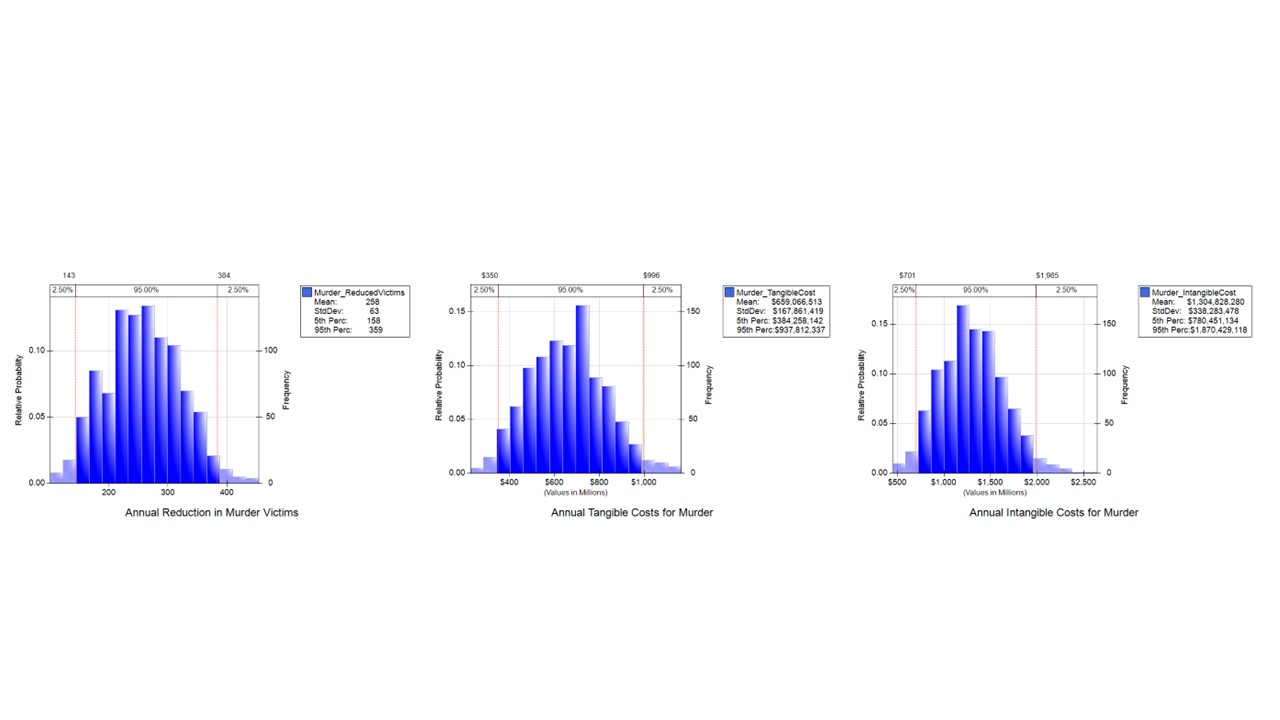


Supplementary Figure 3. Monte Carlo simulation for key outcomes of reduction in victims (left histogram), tangible cost savings (center histogram), and intangible cost savings (right histogram) for murder cases per year. Accompanying sensitivity analyses are shown in Supplementary Figure 7.


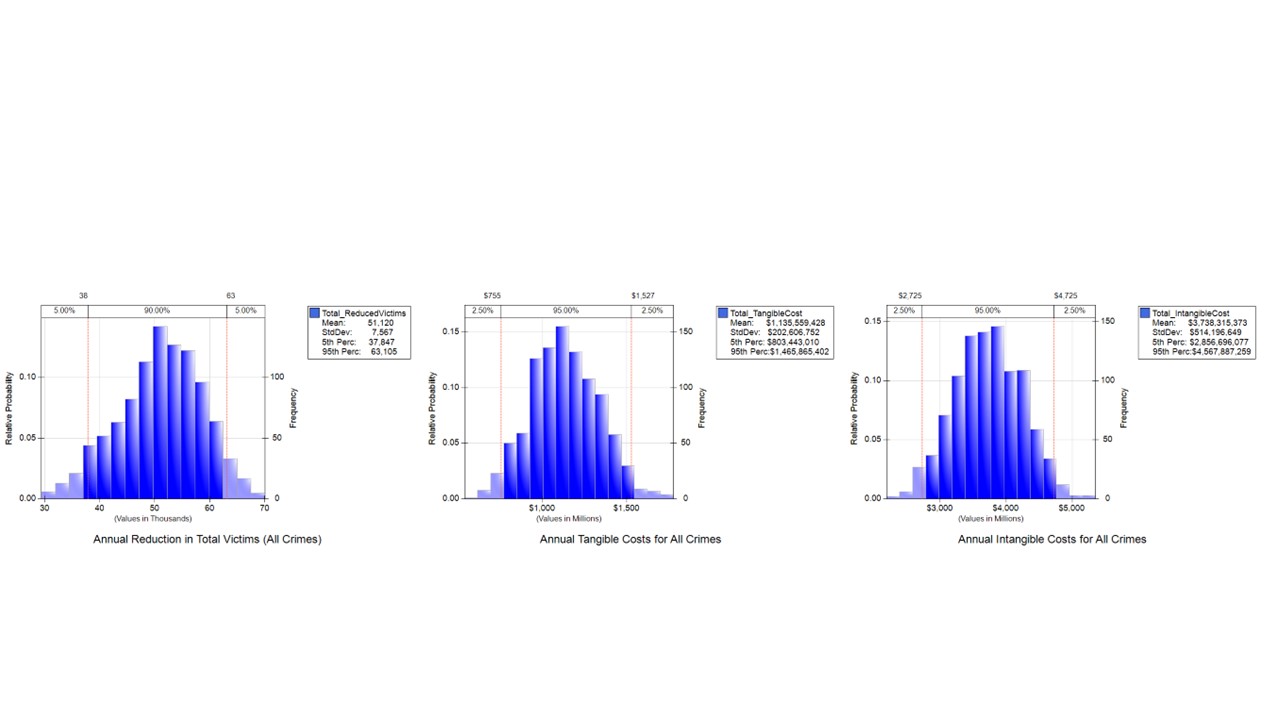


Supplementary Figure 4. Monte Carlo simulation for key outcomes of reduction in victims (left histogram), tangible cost savings (center histogram), and intangible cost savings (right histogram) for all crime cases combined per year. Accompanying sensitivity analyses are shown in Supplementary Figure 8.


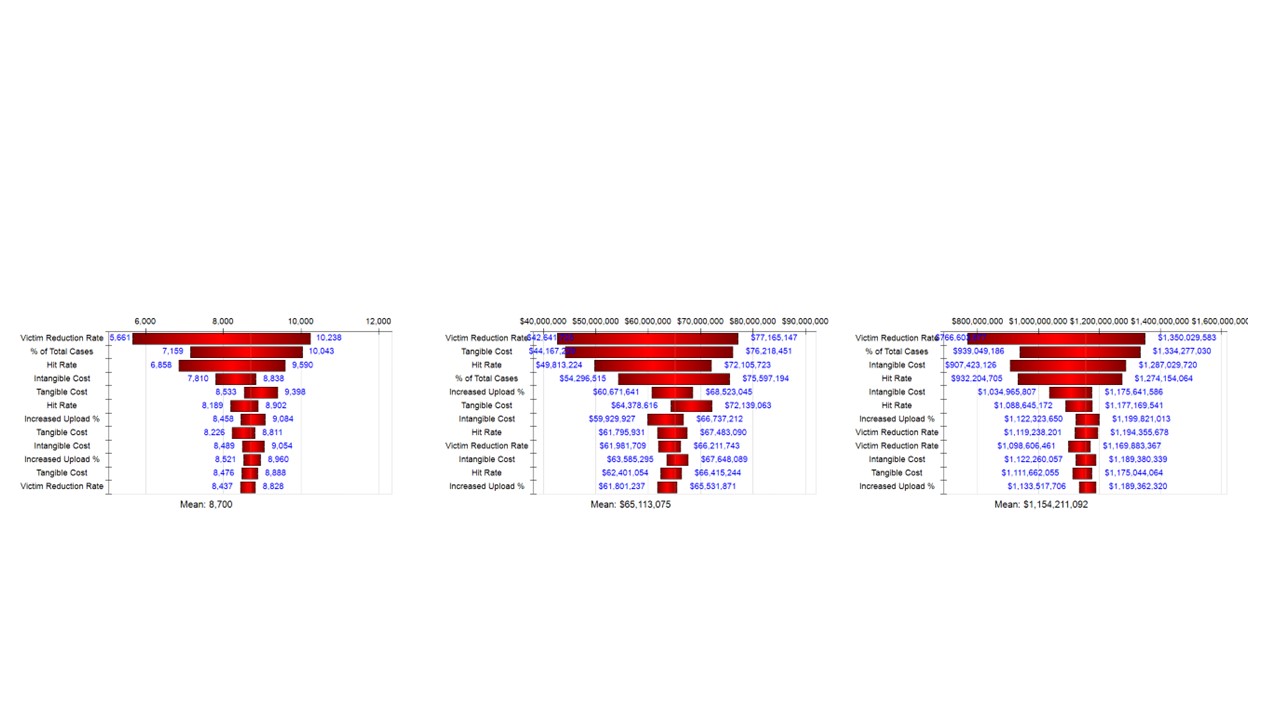


Supplementary Figure 5. Monte Carlo simulation sensitivity analyses for key outcomes of reduction in victims (left histogram), tangible cost savings (center histogram), and intangible cost savings (right histogram) for sexual assault cases per year.


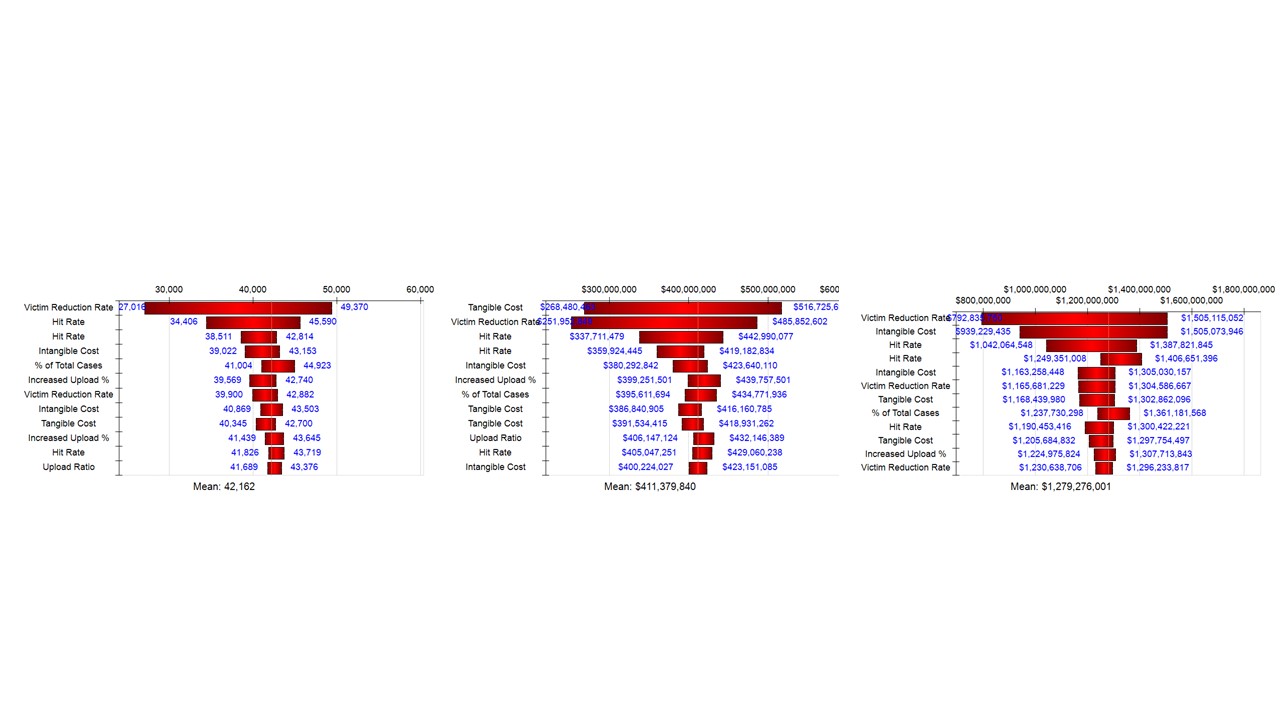


Supplementary Figure 6. Monte Carlo simulation sensitivity analyses for key outcomes of reduction in victims (left histogram), tangible cost savings (center histogram), and intangible cost savings (right histogram) for other crimes per year.


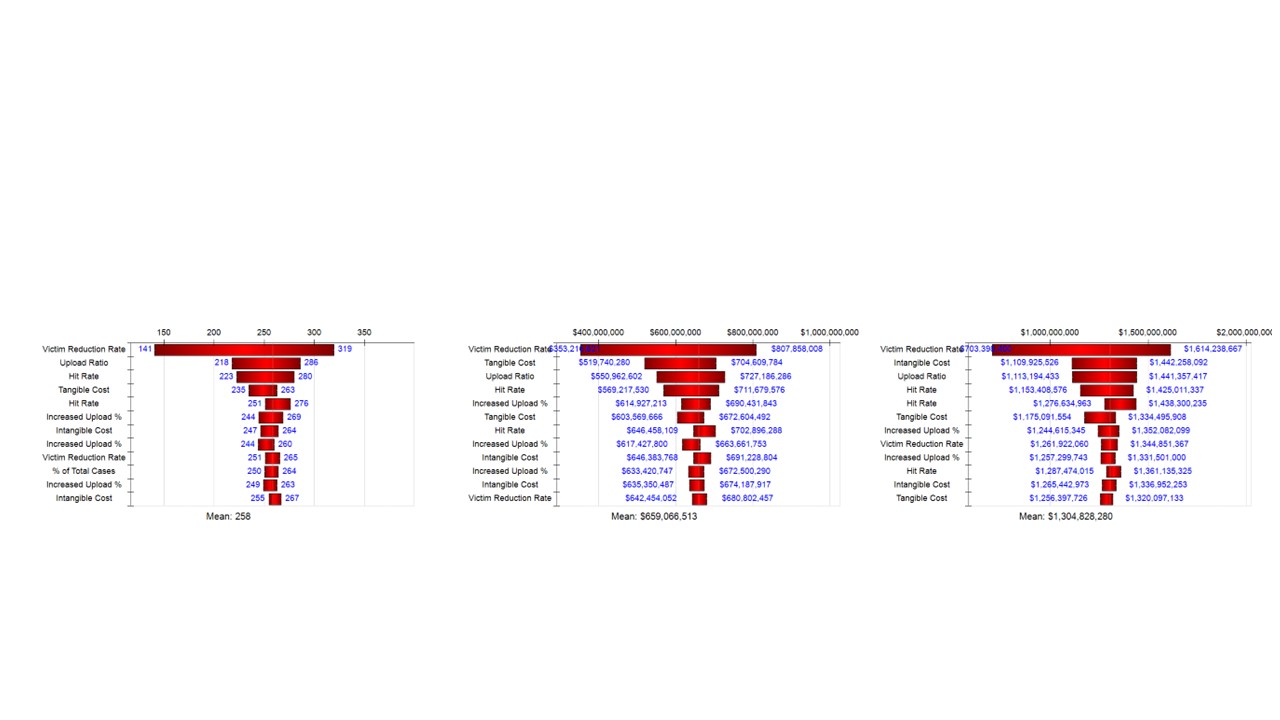


Supplementary Figure 7. Monte Carlo simulation sensitivity analyses for key outcomes of reduction in victims (left histogram), tangible cost savings (center histogram), and intangible cost savings (right histogram) for murder cases per year.


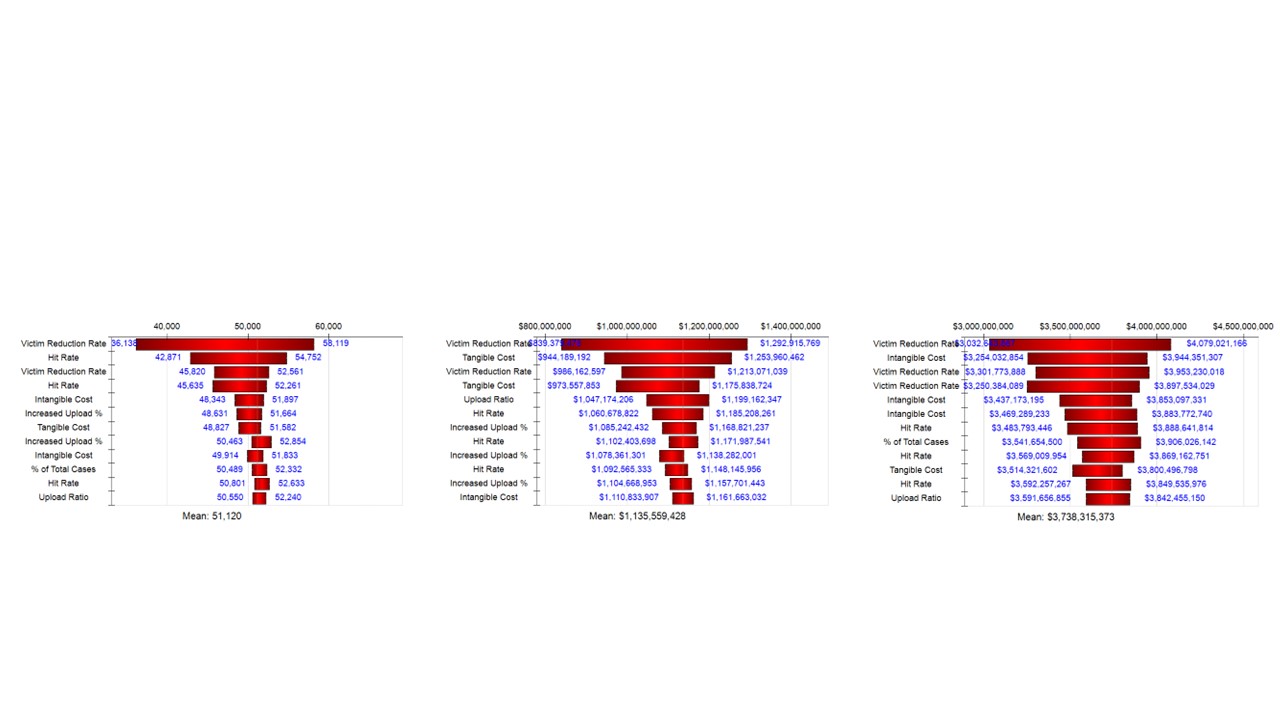


Supplementary Figure 8. Monte Carlo simulation sensitivity analyses for key outcomes of reduction in victims (left histogram), tangible cost savings (center histogram), and intangible cost savings (right histogram) for all crime cases combined per year.
